# Cerebrospinal fluid metabolite alterations in patients with different etiologies, diagnoses, and prognoses of disorders of consciousness

**DOI:** 10.1002/brb3.3070

**Published:** 2023-07-08

**Authors:** Long Xu, Qianqian Ge, Hezhen Lu, Yutong Zhuang, Xiaoli Geng, Xueling Chen, Xiaoyan Liu, Haidan Sun, Zhengguang Guo, Jiameng Sun, Feng Qi, Xia Niu, Aiwei Wang, Wei Sun, Jianghong He

**Affiliations:** ^1^ Department of Neurosurgery Beijing Tiantan Hospital Capital Medical University Beijing China; ^2^ Department of Neurosurgery China National Clinical Research Center for Neurological Diseases (NCRC‐ND) Beijing China; ^3^ Department of clinical laboratory China‐Japan Union Hospital of Jilin University Changchun China; ^4^ Core Instrument Facility, Institute of Basic Medical Sciences, Chinese Academy of Medical Sciences, School of Basic Medicine Peking Union Medical College Beijing China

**Keywords:** biomarkers, cerebrospinal fluid, consciousness disorders, metabolomics

## Abstract

**Introduction:**

Medical management of disorders of consciousness (DoC) is a growing issue imposing a major burden on families and societies. Recovery rates vary widely among patients with DoC, and recovery predictions strongly influence decisions on medical care. However, the specific mechanisms underlying different etiologies, consciousness levels, and prognoses are still unclear.

**Methods:**

We analyzed the comprehensive cerebrospinal fluid (CSF) metabolome through liquid chromatography‐mass spectrometry. Metabolomic analyses were used to identify the metabolic differences between patients with different etiologies, diagnoses, and prognoses.

**Results:**

We found that the CSF levels of multiple acylcarnitines were lower in patients with traumatic DoC, suggesting mitochondrial function preservation in the CNS, which might contribute to the better consciousness outcomes of these patients. Metabolites related to glutamate and GABA metabolism were altered and showed a good ability to distinguish the patients in the minimally conscious state and the vegetative state. Moreover, we identified 8 phospholipids as potential biomarkers to predict the recovery of consciousness.

**Conclusions:**

Our findings shed light on the differences in physiological activities underlying DoC with different etiologies and identified some potential biomarkers used for DoC diagnosis and prognosis.

## Graphical abstract

The cerebrospinal fluid metabolism alterations of disorders of consciousness have been reveled for the first time, which promote the understanding of the pathogenesis. Plenty of biomarkers contribute to diagnosis and prognosis have been identified.

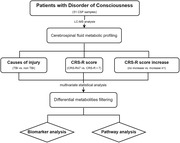

## 1 INTRODUCTION

The term disorders of consciousness (DoC) refers to an altered conscious state when patients show dysfunctions of arousal and awareness regulation (Bai et al., 2021), and it is an important but not fully understood entity in neurology. The progress in the treatment of critically ill neurological patients has lowered the mortality rate of patients with severe brain impairment caused by traumatic brain injury (TBI), anoxic/hypoxic encephalopathy, or stroke, and more patients with DoC are surviving, yet this has imparted a major economic burden on families and societies (Weiss et al., 2007).

Acute severe brain injury leads to coma, a deep unconscious state in which the patient shows no signs of consciousness or a sleep‐wake cycle (Wijnen et al., 2007). Following the recovery of neurological functions, some of these patients will develop a vegetative state (VS) in which they show a sleep‐wake cycle but they cannot communicate or respond to environmental stimuli (von Wild et al., 2012). If the recovery continues, the patients who show fluctuating but definite signs of responses to external stimuli will be defined as being in a minimally conscious state (MCS) (Giacino et al., 2002). The Coma Recovery Scale—Revised (CRS‐R) (Giacino et al., 2004) is the most widely used and the gold standard behavioral scale to assess the conscious state of patients with DoC. Some patients may remain in a VS or MCS for the rest of their lifespan, while others gain full recovery of consciousness and responsiveness. Recovery from DoC to consciousness occurs differently from patient to patient and varies according to etiology (e.g., 52% of traumatic and 15% of nontraumatic VS patients recover to consciousness (Multi‐Society Task Force on PVS, 1994a, 1994b). Since these differences are important for decisions on the medical care of patients with DoC, research is needed to understand what underlies the differences.

Metabolomics is a novel approach that investigates the global metabolite profile in biofluids, and it provides valuable insights into metabolic changes and etiologies during illness. Improvements in liquid chromatography‐mass spectrometry (LC‐MS) have led to great advances in metabolomic analyses (Cui et al., 2018). The metabolite profile in cerebrospinal fluid (CSF) can serve as a direct indicator of biological process variations in the brains of patients with DoC when it is difficult to differentiate these processes based on patient clinical manifestations (Peng et al., 2020). LC‐MS has been widely applied in research on neurological diseases. Through LC‒MS‐based metabolomic analyses, alterations in amino acid, fatty acid, and energy metabolism after TBI have been found (Chitturi et al., 2018); the pathophysiology of vascular cognitive impairment was determined to be caffeine metabolism and the tricarboxylic acid cycle (Peng et al., 2020). Abnormal metabolism associated with other neurological disorders, including ischemic stroke (Au, 2018), brain tumors (Pandey et al., 2017), and neurodegenerative diseases (Luan et al., 2019), has also been studied. However, only one study focused on the metabolome changes in patients with DoC (Yu et al., 2021), and in that study, blood samples were used to identify the altered metabolites of the patients with DoC compared with healthy controls as well as to identify biomarkers that distinguish a vegetative state (VS) from a minimally conscious state (MCS).

Our study focused on the differences among patients with DoC in three dimensions: etiology, diagnosis, and prognosis. We utilized CSF samples to reflect the subtle changes in brain metabolism. CSF samples from 51 patients were collected and were compared according to CRS‐R score, cause of injury, and whether there was an improvement after 3 months. After profiling the global CSF metabolite profile of patients with DoC through an LC‐MS approach, further analyses were used to discover the metabolites differentiating each group and to speculate on potential biomarkers for assessing the conscious state or evaluating prognosis. Applying this metabolomic method to detect DoC will provide new insights into the diagnosis and prognosis of DoC, as well as a further understanding of the metabolic mechanisms underlying conscious recovery.

## 2 MATERIALS AND METHODS

### 2.1 Patients

This study was approved by the Ethics Committee of Beijing Tiantan Hospital, Capital Medical University. All patients provided informed consent before participating in this study. A total of 51 DoC patients were enrolled in Beijing Tiantan Hospital. All patients underwent cranial computerized tomography (CT) scans or brain magnetic resonance imaging (MRI) examinations to clarify the causes of DoC (24 patients caused by TBI, 23 patients caused by intracranial hemorrhages, and 4 patients caused by hypoxic‐ischemic encephalopathy). Their level of consciousness was assessed by experienced physicians using the CRS‐R. CSF samples were collected by trained study personnel in the morning after an overnight fast before clinical treatment and stored at −80°C. None of the patients were in an acute infection stage. The prognosis information, also evaluated by CRS‐R, was collected 3 months after different treatments. All the patients were grouped by the etiologies (TBI vs. non‐TBI), the diagnoses (VS vs. MCS), and prognoses (with or without an increase in the CRS‐R score 3 months after treatment).

### 2.2 Sample preparation

Acetonitrile (200 μL) was added to each CSF sample (200 μL), and the mixture was then vortexed for 30 s and centrifuged at 14,000 × *g* for 10 min. The supernatant was vacuum‐dried and reconstituted with 200 μL 2% acetonitrile. The metabolites were further separated from larger molecules using a 10 kDa molecular weight cutoff ultracentrifugation filter (Millipore Amicon Ultra, MA) before being transferred to an automatic sampler.

### 2.3 Quality control

A pooled sample prepared by mixing aliquots of all CSF samples was used as a quality control (QC) sample. The QC samples were randomly injected throughout the analytical run to evaluate the stability and repeatability of LC‐MS.

### 2.4 LC‐MS analysis

Ultra‐performance LC‐MS analyses of samples were conducted using a Waters ACQUITY H‐class LC system coupled with a Triple TOF 5600 mass spectrometer (AB SCIEX, MA, USA). Metabolites were separated on a Waters HSS C18 column (3.0 × 100 mm, 1.7 μm) with a 17‐min gradient at a flow rate of 0.3 mL/min. Mobile phase A was 0.1% formic acid in H2O, and mobile phase B was acetonitrile. The gradient was set as follows: 0−2 min, 2% solvent B; 2−5 min, 2−55% solvent B; 5−15 min, 55−100% solvent B; 15−20 min, 100% solvent B; 20−20.1 min, 100−2% solvent B; 20.1−29 min, 2% solvent B. The column temperature was set at 50°C. All samples were full scan analyzed from 50 to 1200 *m*/*z*. The full‐scan accumulation time was 0.25 s, the MS/MS accumulation time was 0.1 s, GAS1 and GAS2 were 55, the temperature was 550°C, the ionization spray voltage was 4500 V, and the MS/MS scan collision energy was 35. The top 100 precursors of the full scan were selected for the MS/MS analysis. The dynamic exclusion time was 5 s.

### 2.5 Data processing

Raw data files were processed by Progenesis QI (Waters, Milford, MA, USA) software. Data preprocessing (missing value estimation, log transformation, and Pareto scaling) was carried out to make features more comparable to using Metaboanalyst 5.0 (http://www.metaboanalyst.ca). Variables missed in 50% or more of the samples were deleted without further analysis. Nonparametric tests (Wilcoxon rank‐sum test) were used to evaluate the significance of variables. False‐discovery rate (FDR) correction was used to estimate the probability of false positives and to correct for multiple hypothesis testing. Principal component analysis (PCA) and orthogonal partial least squares discriminant analysis (OPLS‐DA) were performed using SIMCA 14.0 (Umetrics, Sweden) software. The conditions of the differential variables were set as follows: (1) fold‐change (FC) ≥ 1.5 and (2) the variable importance plot (VIP) value obtained from OPLS‐DA was above 1.0. Exploratory ROC analysis and external biomarker validation were performed using the “Biomarker discovery” module of the MetaAnalyst 5.0 platform.

## 3 RESULTS

### 3.1 Patients

**FIGURE 1:**
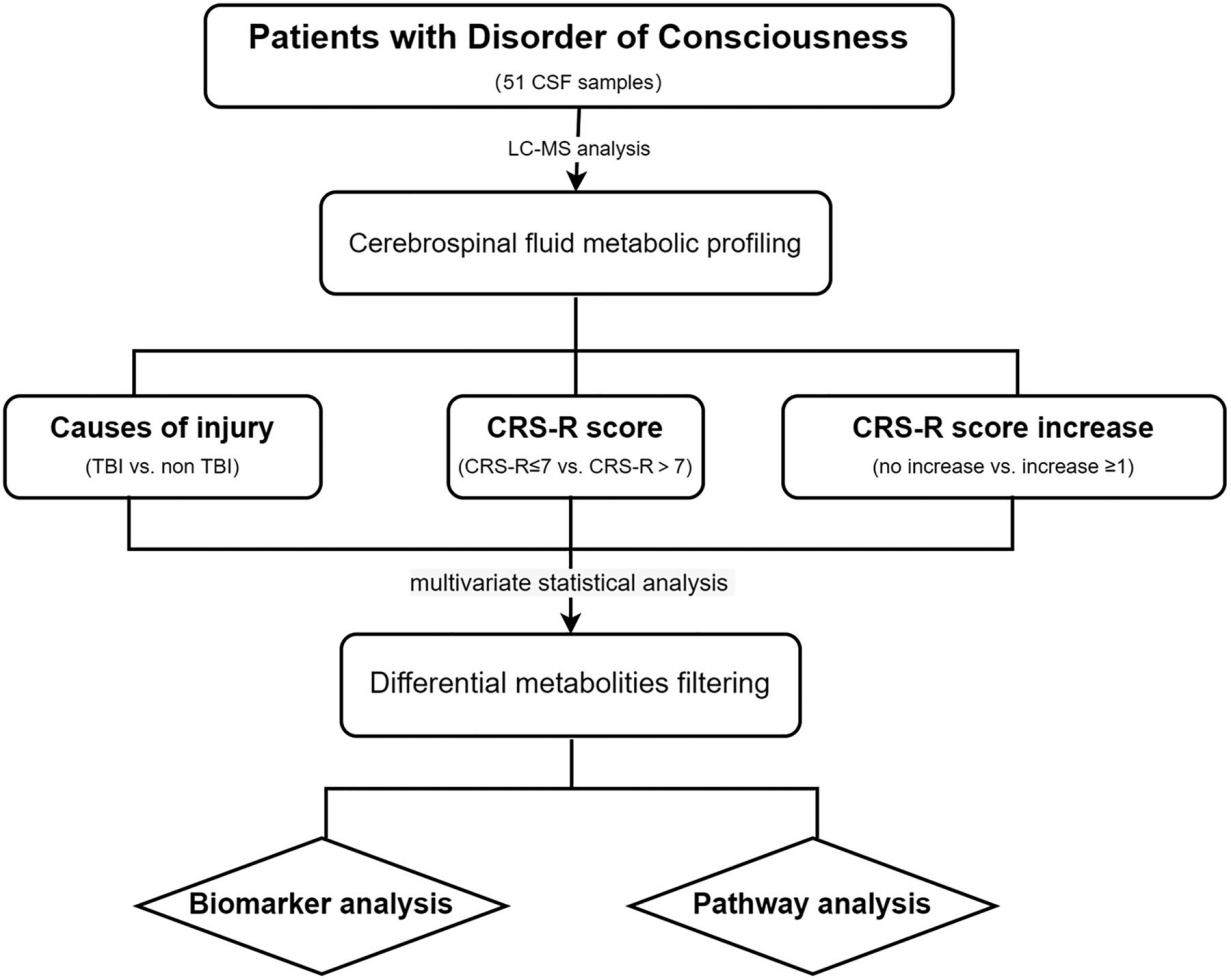
Study design of CSF metabolomics in DoC patients. CSF: cerebrospinal fluid, CRS‐R: Coma Recovery Scale—Revised, TBI: traumatic brain injury.

We aimed to identify different metabolic pathways underlying DoC caused by different etiologies, contrasting metabolic profiles between patients with different CRS‐R scores, and potential biomarkers for distinguishing patients with the capacity to regain consciousness. Therefore, 51 patients (34 VS and 17 MCS) who suffered from prolonged DoC were enrolled in our study. Three months after different treatments, 19 patients had an increased CRS‐R score, and no patient died or was lost to follow‐up. At the final evaluation, 24 patients were in VS, 22 patients were in MCS, and 5 patients emerged from MCS. The workflow of the present study is shown in Figure 1. The baseline clinical information of all enrolled patients is shown in Table 1. No significant differences in any demographic characteristics or laboratory indicators (blood and CSF routine examinations, liver and kidney function indicators, and inflammatory indicators), except for the principle of grouping, were observed between groups. CSF samples were used for the subsequent LC‐MS analysis. The data quality was assessed by calculating the coefficient of variation (CV) of the protein abundance in the QC sample. Pearson's correlation coefficients were all greater than 0.8 (Figure S1), showing good technical reproducibility.

**TABLE 1:** Clinical characteristics of patients with DoC.

	Etiology		Diagnosis		Increase	
Characteristics	TBI	Non‐TBI	VS	MCS	0	≥1
Patients (*n*)	24	27	34	17	32	19
Male/female (*n*)	18/6	17/10	24/10	11/6	22/10	13/6
Age (years)	53.9 ± 21.6	45.3 ± 19.4	50.3 ± 17.5	48.9 ± 13.9	53.2 ± 14.1	48 ± 23.7
Time since brain injury (months)	7.6 ± 7.4	5.0 ± 3.8	5.8 ± 4.5	6.9 ± 8.0	6.6 ± 6.9	5.6 ± 3.7
TBI/non‐TBI (*n*)	24/24	0/24	17/17	7/10	16/16	18/19
CRS‐R	6.4 ± 3.1	7.3 ± 3.7	4.8 ± 1.4	11.0 ± 2.2	6.3 ± 3.5	7.8 ± 3.0
Increase	0.7 ± 1.1	0.6 ± 0.9	0.7 ± 1.0	0.6 ± 0.8	0	1.6 ± 0.8
WBC	3.0 ± 2.3	4.8 ± 3.3	3.9 ± 3.2	3.5 ± 2.3	4.1 ± 2.2	3.0 ± 3.9
Pro	57.5 ± 56.5	40.0 ± 12.3	48.4 ± 41.3	54.3 ± 51.3	50.0 ± 37.8	51.4 ± 57.9
Glu	4.1 ± 0.9	4.3 ± 0.7	4.2 ± 0.7	4.1 ± 1.1	4.1 ± 0.8	4.2 ± 1.0
Lac	2.0 ± 0.6	2.2 ± 0.3	2.2 ± 0.4	1.9 ± 0.6	2.1 ± 0.6	2.1 ± 0.4

Continuous variables are expressed as mean ± standard deviation (SD); *Increase* refers to an increase in CRS‐R scores 3 months after treatment.

TBI: traumatic brain injury; CRS‐R: Coma Recovery Scale—Revised score.

### 3.2 Metabolic differences between different etiologies of DoC

**FIGURE 2:**
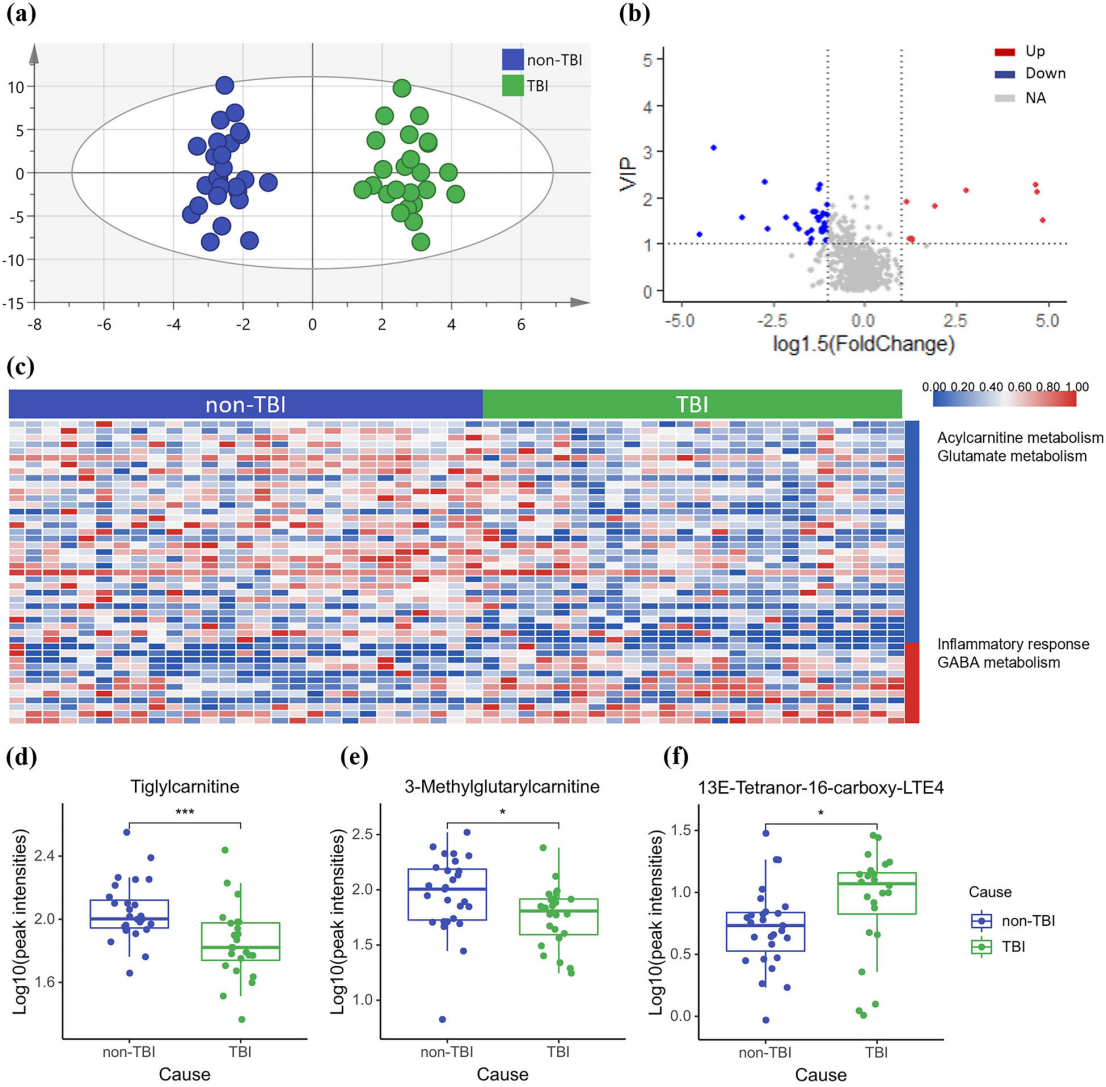
Analysis of CSF metabolomic of TBI‐induced DoC and non‐TBI‐induced DoC. (a) OPLS‐DA model based on patients’ CSF for classification of TBI‐induced and non‐TBI‐induced DoC; (b) volcano plot for filtering differential metabolites (VIP value ≥1, fold‐change ≥1.5); (c) heatmap of differential metabolites in the two groups and the differential metabolic pathways listed on the right side (blue indicate relative lower and red indicate relative higher in TBI‐induced DoC group); (d–f) box plots for relative intensities of tiglylcarnitine, 3‐methylglutarylcarnitine, and 13E‐tetranor‐16‐carboxy‐LTE4 in non‐TBI‐induced and TBI‐induced DoC.

**FIGURE 3:**
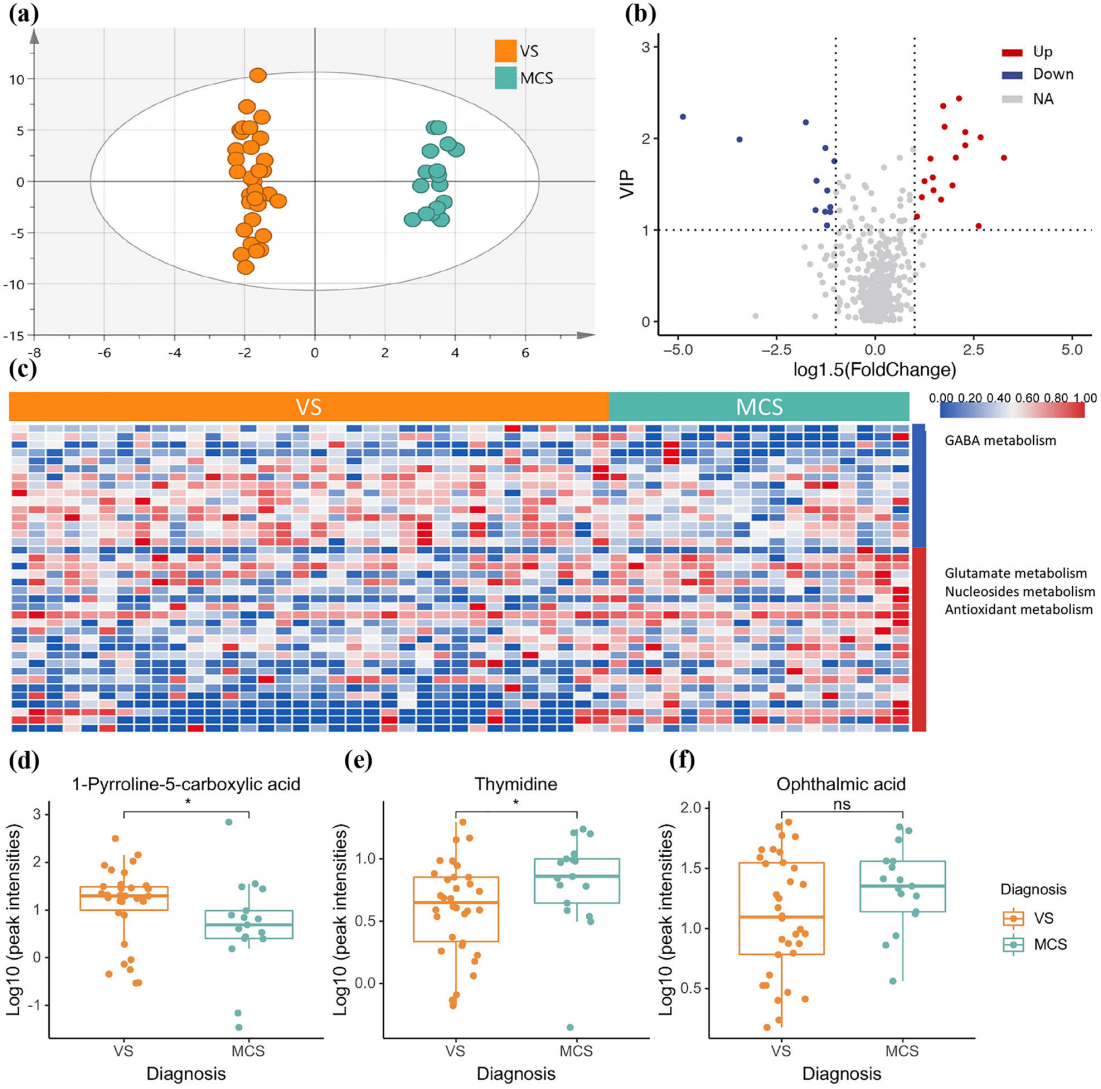
Analysis of CSF metabolomic of DoC patients with different diagnoses. (a) OPLS‐DA model based on patients’ CSF for classification of patients with CRS‐R scores less than or equal to 7 and patient with CRS‐R scores greater than 7; (b) volcano plot for filtering differential metabolites (VIP value ≥1, fold‐change ≥1.5); (c) heatmap of differential metabolites in the two groups and the differential metabolic pathways listed on the right side (blue indicate relative lower and red indicate relative higher in the group with higher CRS‐R scores); (d–f) box plots for relative intensities of 1‐pyrroline‐5‐carboxylic acid, thymidine, and ophthalmic acid in DoC patients with different CRS‐R scores.

An unsupervised PCA and a supervised OPLS‐DA model were created to show the preliminary differences in the metabolic profiles of DoC caused by different etiologies. The results are shown in Figures S1a and 2a. The CV‐ANOVA *p* value of the OPLS‐DA model was less than .01, a significant difference between the two groups was also shown by the score plots. The variable importance in projection (VIP) value in the OPLS‐DA model (Figure S2c) combined with the fold‐change was used to define the differential metabolites (Figure 2b). Forty‐five differential metabolites between the different etiologies were identified based on a VIP value ≥1 and a fold‐change ≥ 1.5 (Figure 2c). After further investigation of the functions of the differential metabolites, metabolites related to acylcarnitine (AC) and glutamate metabolism were found to be decreased in TBI‐induced DoC compared with non‐TBI‐induced DoC. Metabolites related to the inflammatory response and GABA metabolism were increased in the TBI‐induced DoC group compared with the non‐TBI‐induced DoC group. The relative contents of the representative metabolites of these altered metabolic pathways are presented in Figure 2. Tiglylcarnitine (Figure 2d) and 3‐methylglutarylcarnitine (Figure 2e) were significantly lower in the TBI‐induced DoC group, while the inflammatory metabolite 13E‐tetranor‐16‐carboxy‐LTE4 (Figure 2f) was significantly elevated. To further explore the differentiating ability of each metabolite, a ROC curve was created for each molecule. According to the ROC curve, 5‐methyl‐THF showed good performance in differentiating the TBI‐induced DoC group from the non‐TBI‐induced DoC group, with an AUC above 0.8. The other 11 endogenous metabolites (Table 2) with an AUC above 0.7 also showed a potential ability to differentiate DoC due to different etiologies.

**TABLE 2:** Biomarkers between different etiologies of DoC.

Biomarkers	AUC	VIP	FC	*p*
5‐Methyl‐THF	0.82	4.93	0.02	<.01
Tiglylcarnitine	0.78	1.80	0.66	.02
3‐hydroxy‐4‐(3‐hydroxyphenyl)−1‐methyl‐5‐phenylpiperidine‐2,6‐dione	0.76	2.33	0.33	.01
Cotinine glucuronide	0.74	1.63	0.66	.23
1‐Pyrroline‐5‐carboxylic acid	0.73	5.26	1533.43	.38
3‐Methyldioxyindole	0.73	3.53	0.03	.33
L‐gamma‐glutamyl‐L‐isoleucine	0.73	2.14	3.06	<.01
L‐Glutamate	0.71	3.06	0.19	.10
Isoxanthopterin	0.71	1.55	0.59	.01
13E‐Tetranor‐16‐carboxy‐LTE4	0.71	1.81	2.18	.02
3‐Methylglutarylcarnitine	0.70	1.65	0.64	.01
Asp Gly Pro Pro	0.70	1.59	0.62	.01

AUC: area under curve; VIP: variable importance in projection; FC: fold‐change.

### 3.3 Metabolic differences between different diagnoses

Similarly, pilot differential analysis utilizing PCA (Figure S3a) and OPLS‐DA (Figure 3a) models were performed to discriminate the overall difference between two groups with different CRS‐R scores. The score plot of OPLS‐DA from the two groups showed an apparent differentiation, and the CV‐ANOVA *p* value of the OPLS‐DA model was less than 0.01. The global distribution of each metabolite is presented in the volcano plot (Figure 3b), for which the VIP value (Figure S3c) and fold‐change were adopted as the coordinate axes. A total of 38 differential metabolites were identified for the two groups with different CRS‐R scores based on the principle of a VIP value ≥1 and a fold‐change ≥1.5. From the heatmap of these metabolites (Figure 3c), it is not difficult to conclude that metabolites related to GABA metabolism were downregulated, while metabolites related to nucleoside metabolism and glutamate metabolism were upregulated in the group with a higher CRS‐R score. For the representative metabolites, 1‐pyrroline‐5‐carboxylic acid (Figure 3d) was significantly decreased, thymidine (Figure 3e) was significantly increased, and ophthalmic acid (Figure 3f) was relatively but not significantly increased in the higher CRS‐R score group. The differentiation ability of each differential metabolite was further explored using ROC curve analysis. ROC curve analysis showed that 9 metabolites (Table 3) had good diagnostic values for patients with different CRS‐R scores, with AUC values above 0.7.

**TABLE 3:** Biomarkers between DoC patients with different diagnoses.

Biomarkers	AUC	VIP	FC	*p*
Proline betaine	0.81	6.13	0.01	.14
Sphingofungin A	0.79	4.49	132.05	.26
PC(16:0/18:2(9Z,12Z))	0.79	5.74	<0.01	.56
Mulberrofuran T	0.78	4.70	112.35	.85
PE(16:0/22:2(13Z,16Z))	0.74	5.84	<0.01	.41
PC(16:0/22:5(4Z,7Z,10Z,13Z,16Z))	0.73	2.13	0.49	.22
Glycochenodeoxycholic acid 3‐glucuronide	0.72	2.35	0.50	.82
Cyclomammein	0.72	1.75	1.52	.01
Calendic acid	0.71	1.20	1.67	.05

AUC: area under curve; VIP: variable importance in projection; FC: fold‐change.

### 3.4 Metabolic differences between different prognoses of DoC

**FIGURE 4:**
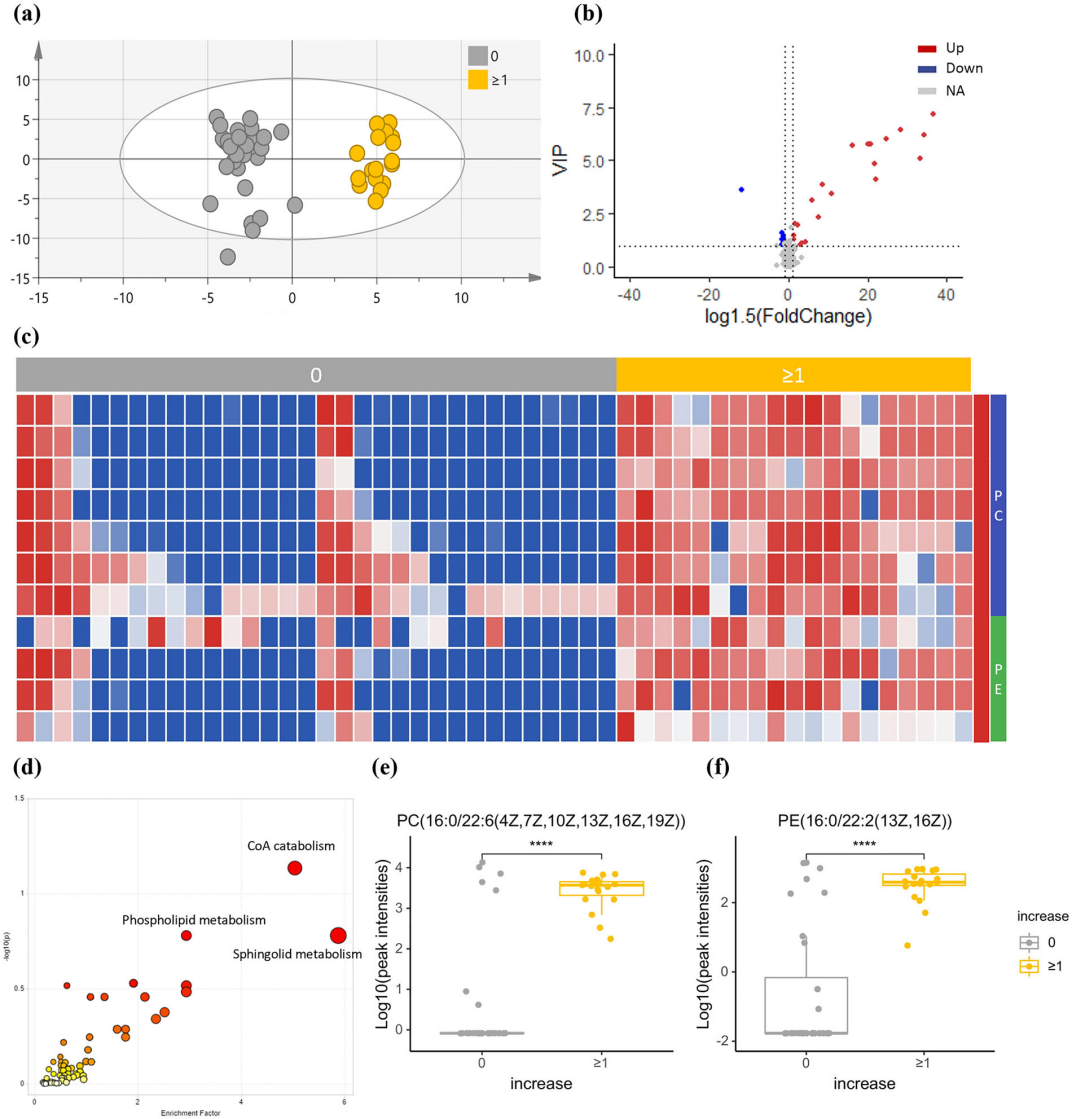
Analysis of CSF metabolomic of DoC patients with different prognoses. (a) OPLS‐DA model based on patients’ CSF for classification of patients with and without improvement after 3 months; (b) volcano plot for filtering differential metabolites (VIP value ≥1, fold‐change ≥1.5); (c) heatmap of differential phospholipids in the two groups which were all higher in improved group (red block) and the categories of phospholipids are shown in different color blocks on the right; (d) biological pathway of differential metabolites; (e, f) box plots for relative intensities of representative PC and PE in DoC patients with different prognoses.

Distinguishing patients with the capacity to regain consciousness from others was further performed to explore metabolic differences. PCA showed slight discrimination between the two groups with different outcomes (Figure S4a). Furthermore, an OPLS‐DA model achieved significant discrimination (*p* < .01) (Figure 4a), with 37 features (Figure S4c) contributing to group differentiation (VIP value ≥1). Among these features, 28 metabolites were selected as differential metabolites for further investigation. A heatmap of these differential metabolites was plotted (Figure S4d). Compared with patients showing no sign of recovery from DoC, metabolic pathways concerning glycerophospholipids, GABA, and anti‐inflammation were upregulated, and metabolic pathways concerning oxidative stress were downregulated in the group with improved performance. Eleven of the differential metabolites were phospholipids (Figure 4c), composed of 7 phosphatidylcholines (PCs) and 4 phosphatidylethanolamines (PEs), which were all significantly elevated in the group with an increase in their CRS‐R scores. Functional analysis of the entire metabolic profile (Figure 4d) also showed significant alterations in phospholipid metabolism. The relative contents of representative metabolites of PCs (Figure 4e) and PEs (Figure 4f) are shown in Figure 4. ROC curve analysis showed that 10 of the phospholipids (Table 4) had good diagnostic values for predicting the prognosis of DoC, with AUC values above 0.7. PC (18:0/22:6(4Z,7Z,10Z,13Z,16Z,19Z)) and PC (20:5(5Z,8Z,11Z,14Z,17Z)/P‐18:1(11Z)) showed superior differentiating ability, with AUC values above 0.9, and thus might serve as reliable biomarkers for the prognosis of DoC.

**TABLE 4:** Biomarkers between different prognoses of DoC.

Biomarkers	AUC	VIP	FC	*p*
PC(18:0/22:6(4Z,7Z,10Z,13Z,16Z,19Z))	0.92	3.88	0.03	<.01
PC(20:5(5Z,8Z,11Z,14Z,17Z)/P‐18:1(11Z))	0.91	5.75	<0.01	<.01
PE(20:4(5Z,8Z,11Z,14Z)/22:4(7Z,10Z,13Z,16Z))	0.89	3.14	0.09	<.01
PE(20:3(5Z,8Z,11Z)/22:6(4Z,7Z,10Z,13Z,16Z,19Z))	0.88	7.19	<0.01	<.01
PC(16:0/18:2(9Z,12Z))	0.88	6.44	<0.01	.06
PC(16:0/22:6(4Z,7Z,10Z,13Z,16Z,19Z))	0.88	5.78	<0.01	.01
PE(16:0/22:2(13Z,16Z))	0.87	6.04	<0.01	<.01
PC(16:0/18:1(11Z))	0.83	6.18	<0.01	.04
PE(15:0/18:3(9Z,12Z,15Z))	0.76	2.32	0.05	.01
PC(16:0/22:5(4Z,7Z,10Z,13Z,16Z))	0.75	4.08	<0.01	.18

AUC: area under curve; VIP: variable importance in projection; FC: fold‐change.

## 4 DISCUSSION

Our results revealed that the CSF metabolome differed between patients with distinct DoC etiologies and could be used to differentiate DoC patients with different CRS‐R scores. Moreover, the metabolomic profile could be used to predict the outcomes of patients with DoC after medical treatment according to an increase in CRS‐R scores. Underlying metabolic pathways and potential biomarkers of different etiologies, diagnoses, and prognoses of patients with DoC were identified, and the results provided new insights into the treatment of DoC.

### 4.1 Glutamate and GABA metabolism differs in MCS and VS patients

Similar to Yu et al. (2021), we observed changes in the purine metabolic pathway and lipids between the two groups of patients with different CRS‐R scores. Additionally, we noticed that a few metabolites, including L‐glutamate, ophthalmic acid, gabapentin, and 1‐pyrroline‐5‐carboxylic acid, related to glutamate and GABA metabolism were altered and had a good ability to distinguish these two groups of patients. As the major excitatory and inhibitory neurotransmitters in the central nervous system, glutamate, and GABA have been widely studied in neurological disorders (Onaolapo & Onaolapo, 2021; Petroff, 2002). However, limited research has been done on the relationship between these metabolites and DoC, and further research is needed. In our study, 1‐pyrroline‐5‐carboxylic acid, which can act as a neurotoxin under certain conditions (Deuschle et al., 2004), was found to be higher in patients with lower CRS‐R scores than in those with higher scores. 1‐Pyrroline‐5‐carboxylic acid, an intermediate in glutamate metabolism, accelerates the conversion of glutamate to L‐glutamate gamma‐semialdehyde (Wan et al., 2014) and participates in the negative feedback of GABA to the metabolic flow from L‐ornithine to L‐glutamic acid (Yoneda et al., 1982). Elevated 1‐pyrroline‐5‐carboxylic acid levels in patients with worse clinical presentation might indicate the suppression of the excitatory neurotransmitter glutamate as well as the overactivation of inhibitory GABA, which causes the inhibition of consciousness. On the other hand, the overall functions of the differential metabolites between patients with high versus low CRS‐R scores were dispersive and unclear. The same problems were also present in the research of Jie Yu et al., and most of the metabolites altered in patients with DoC compared to healthy controls did not show statistically significant differences between patients in an MCS and those in a VS. This might result from the limited sample size or the poor ability of the CRS‐R score to distinguish subtle changes within DoC patients (Owen, 2020).

### 4.2 Acylcarnitine metabolism differs in TBI‐induced DoC and non‐TBI‐induced DoC patients

Severe brain injuries leading to DoC are mainly caused by TBI, ischemic stroke, hemorrhagic stroke, or hypoxic‐ischemic encephalopathy. Interestingly, according to previous studies, patients suffering from DoC caused by TBI are more likely to regain consciousness (Kowalski et al., 2021; McCrea et al., 2021). To determine the underlying differences between TBI‐induced DoC and non‐TBI‐induced DoC, we analyzed and compared the global metabolic profiles of these two groups of patients. We observed that a wide range of ACs, including tiglylcarnitine, 3‐methylglutarylcarnitine, 2‐methylbutyroylcarnitine, and butyrylcarnitine, were downregulated in TBI‐induced DoC patients relative to non‐TBI‐induced DoC patients. The general role of ACs is to transport long‐chain fatty acids into the mitochondria for β‐oxidation, and this process also helps in the removal of organic acids (Bremer, 1983). Increased levels of plasma ACs were associated with decreased mitochondrial activity and could activate proinflammatory responses (Jarrell et al., 2020). As an energy‐intensive organ, the importance of ACs in the brain is intuitive. Serum levels of ACs could be used to predict the subsequent changes in cognitive functions of Alzheimer's disease (AD) patients (Huo et al., 2020), and a dramatic increase in AC levels in different brain regions and plasma was also observed after hypoxic‐ischemic brain injury (Dave et al., 2022). In our study, the AC levels in the CSF of TBI‐induced DoC patients were lower than those in other patients with DoC, suggesting the preservation of mitochondrial function in patients with DoC caused by TBI, which might explain the better outcomes of TBI‐induced DoC patients. However, there is evidence showing that AC and carnitine supplementation has beneficial effects on various neurological diseases (Jones et al., 2010), which cannot explain the contradiction between the high level of ACs and poor outcomes of non‐TBI patients with DoC. Interestingly, among the decreased ACs, tiglylcarnitine, 2‐methylbutyroylcarnitine, and butyrylcarnitine were all short‐chain ACs, which is the most abundant group of ACs in the body (Makarova et al., 2019). Short‐chain AC levels have been found to increase significantly during the progression of core depression and anxious depression (Ahmed et al., 2020) but decrease in AD (Horgusluoglu et al., 2022). In an animal experiment, the concentration of short‐chain ACs could be elevated after a 72‐h starvation period (Murakami et al., 1997). The inconsistent results of the changes in short‐chain ACs after different neurological diseases indicate that the changes in the levels of short‐chain ACs imply an altered profile of energy metabolic substrates rather than elevated or depressed energy generation. The study of ACs is an active field of research, while limited research has been conducted on the relationship between ACs and DoC. Our study is the first to reveal the alteration of ACs between TBI‐induced DoC and non‐TBI‐induced DoC patients, and further investigation is needed to understand the role ACs play in DoC.

### 4.3 Lipid metabolism might serve as a reliable biomarker for the prognosis of DoC

Most importantly, we identified biomarkers that had good accuracy for the prediction of patients whose brain networks might be amenable to therapeutic modulations. Whether and which patients with DoC have the potential to recover are major concerns for families and are vital for clinical decision‐making. The development of relevant imaging and electrophysiological techniques can help in discussions about the potential for recovery (Kotchoubey & Pavlov, 2018). However, no single tool accounts for the variance in the outcome of patients with DoC, and economic concerns limit the use of these methods (Edlow et al., 2021). The CSF biomarkers identified in the current study can be integrated into a multimodal approach for the prognostic evaluation of patients with DoC to enhance accuracy. Furthermore, these biomarkers can help us to understand the biological processes underlying the recovery of consciousness and even develop medicines to promote recovery. In the present study, eight of the 11 metabolites with an AUC above 0.8 were phospholipids. Lipids constitute 50% of the brain's dry weight, and the lipid content of the brain is second only to the adipose tissue content (Bruce et al., 2017). Phospholipids are a class of lipids that are crucial to both cell membranes and secondary messengers and are categorized as PCs, PEs, phosphatidylinositols (PIs), and phosphatidylglycerols (PGs). A decrease in phospholipids in serum has been found from 24 h to more than 4 weeks after TBI, while studies on the CSF level of phospholipids were limited to the acute phase of TBI and showed contrasting results (Nessel & Michael‐Titus, 2021). Alterations in brain phospholipid metabolism could also be associated with AD (Kosicek & Hecimovic, 2013). For instance, PI, PE, and PC levels were found to be decreased in postmortem brain tissue from individuals with AD (Prasad et al., 1998; Prasad et al., 1998; Wells et al., 1995). Membrane instability and synaptic loss are thought to be connected with phospholipid alterations (Kosicek & Hecimovic, 2013), which may hinder recovery from DoC. Between the two groups of patients with different outcomes, the levels of 5 PCs were significantly increased in the patients who had an increase in their CRS‐R score after different treatments. PCs contain a choline molecule as the head group; hence, PCs are highly available nutraceuticals to supply choline. The level of PCs is directly linked to the levels of choline and acetylcholine (Javaid et al., 2021). Research on hemorrhagic shock showed that membrane PC depletion was present after shock as the result of cholinergic hyperactivation (Leskova, 2017). Cholinergic hyperactivation is the basis of mitochondrial functional disturbances (Leskova, 2017), which lead to energy deficiency and neuron death. The supplementation of PCs through drugs, including alpha‐glyceryl phosphorylcholine, Fortasyn Connect, or lecithin alone, has shown the potential to slow the progression of AD and dementia and to improve cognition and remyelination after controlled cortical impact injury (Javaid et al., 2021). This evidence indicates that the increase in the CRS‐R score in this group of patients might result from the higher level of PCs; therefore, PC supplementation might be a therapeutic method for DoC. However, these results are very preliminary, and further investigation is needed to verify the potential prognostic and therapeutic value of phospholipids.

## 5 CONCLUSION

In conclusion, we applied a CSF metabolomic approach to identify the metabolic features of DoC patients for the first time. Our results showed markedly different metabolic profiles between patients with different etiologies, CRS‐R scores, and outcomes. Glutamate and GABA metabolism were found to be different in patients with different CRS‐R scores. AC levels were lower in the CSF of patients with TBI‐induced DoC than in those with DoC caused by other brain injuries. Moreover, we identified 8 phospholipids as biomarkers that had a good ability to predict the recovery of consciousness. We also hypothesize that the recovery of consciousness results from the beneficial effects of PCs, and supplementation with PCs may be a therapeutic method for DoC.

However, this study has several limitations. First, the effects of the different therapies the patients received may not have been eliminated given the complex complications of the different patients. We also did not exclude the effect of different diets and other underlying diseases. Second, the size of the sample in our study was relatively small, and we did not enroll healthy controls since the collection of CSF is an invasive procedure. Finally, the prognostic accuracy of the biomarkers we selected still needs to be externally validated and verified through other approaches, including but not limited to in vitro and in vivo experiments.

## AUTHOR CONTRIBUTIONS

L.X., W.S., and J.H. designed the study; Y.Z., X.G., and X.C. recruited the cohort and collected the CSF samples; X.L., H.S., Z.G., J.S., F.Q., X.N., A.W., and W.S. performed the experiments; Q.G. and H.L. analyzed the data; L.X. and Q.G. interpreted the data and wrote the original draft; L.X., Q.G., W.S., and J.H. provided edits to the manuscript. All authors have read and agreed to the published version of the manuscript.

## CONFLICT OF INTEREST STATEMENT

The authors declare no conflict of interest.

### PATIENT CONSENT STATEMENT

Informed consent was obtained from the patients’ legal guardians of all subjects involved in the study.

### PEER REVIEW

The peer review history for this article is available at https://publons.com/publon/10.1002/brb3.3070

## Supporting information

Figure S1. QC samples correlation analysis.
Figure S2. Supplementary analysis of CSF metabolomic of TBI‐induced DoC and non‐TBI‐induced DoC.
Figure S3. Supplementary analysis of CSF metabolomic of DoC patients with different diagnosis.
Figure S4. Supplementary analysis of CSF metabolomic of DoC patients with different prognoses.

## Data Availability

The data supporting this study's findings are available on request from the corresponding author. The data are not publicly available due to privacy or ethical restrictions.
