# Supplementary material for: Cerebrospinal fluid metabolite alterations in patients with different etiologies, diagnoses, and prognoses of disorders of consciousness
Source: Brain Behav. 2023 Jul 8;13(8):e3070. doi: 10.1002/brb3.3070 (PMC10454269; doi:10.1002/brb3.3070)
Supplement: Supplementary file 1 — Figure S1. QC samples correlation analysis. Figure S2. Supplementary analysis of CSF metabolomic of TBI‐induced DoC and non‐TBI‐induced DoC. Figure S3. Supplementary analysis of CSF metabolomic of DoC patients with different diagnosis. Figure S4. Supplementary analysis of CSF metabolomic of DoC patients with different prognoses. [file BRB3-13-e3070-s001.docx]

**
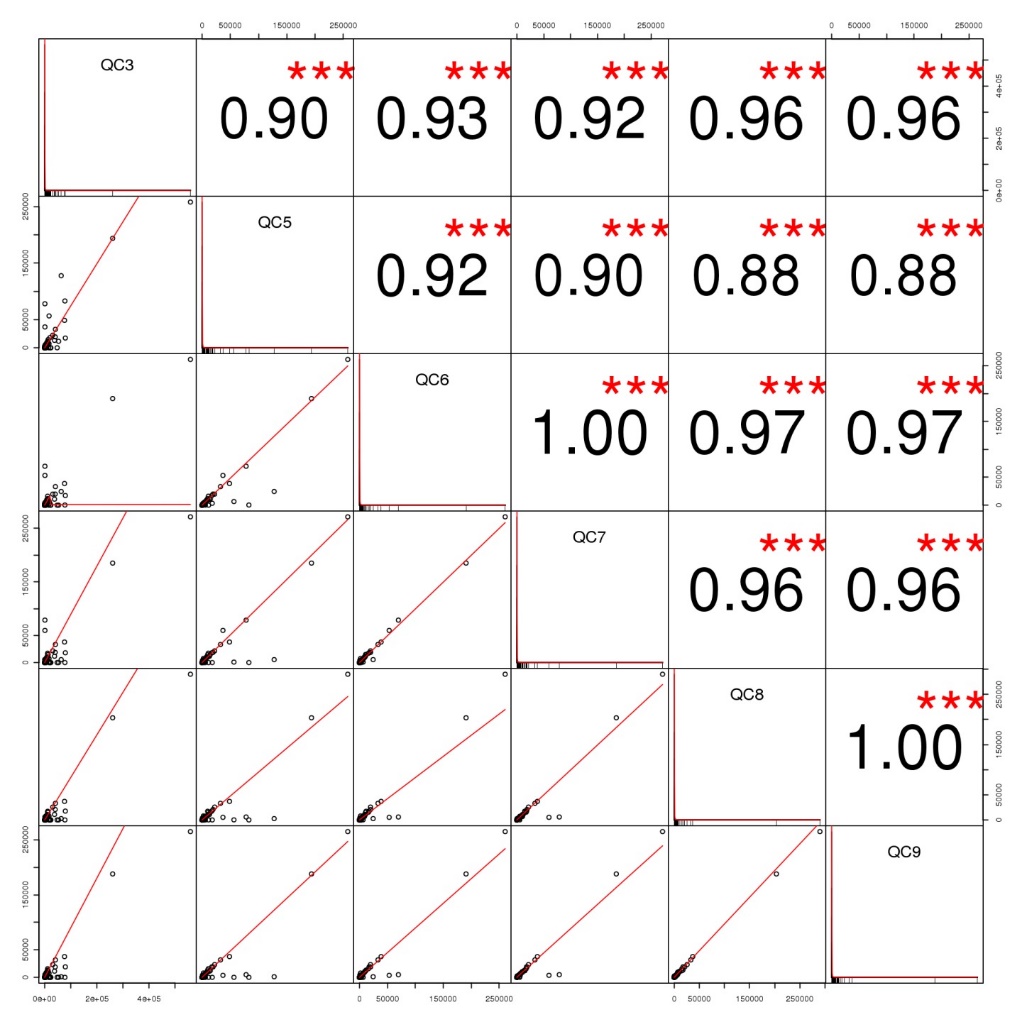
**

**Supporting Figure 1. QC Samples correlation analysis.**


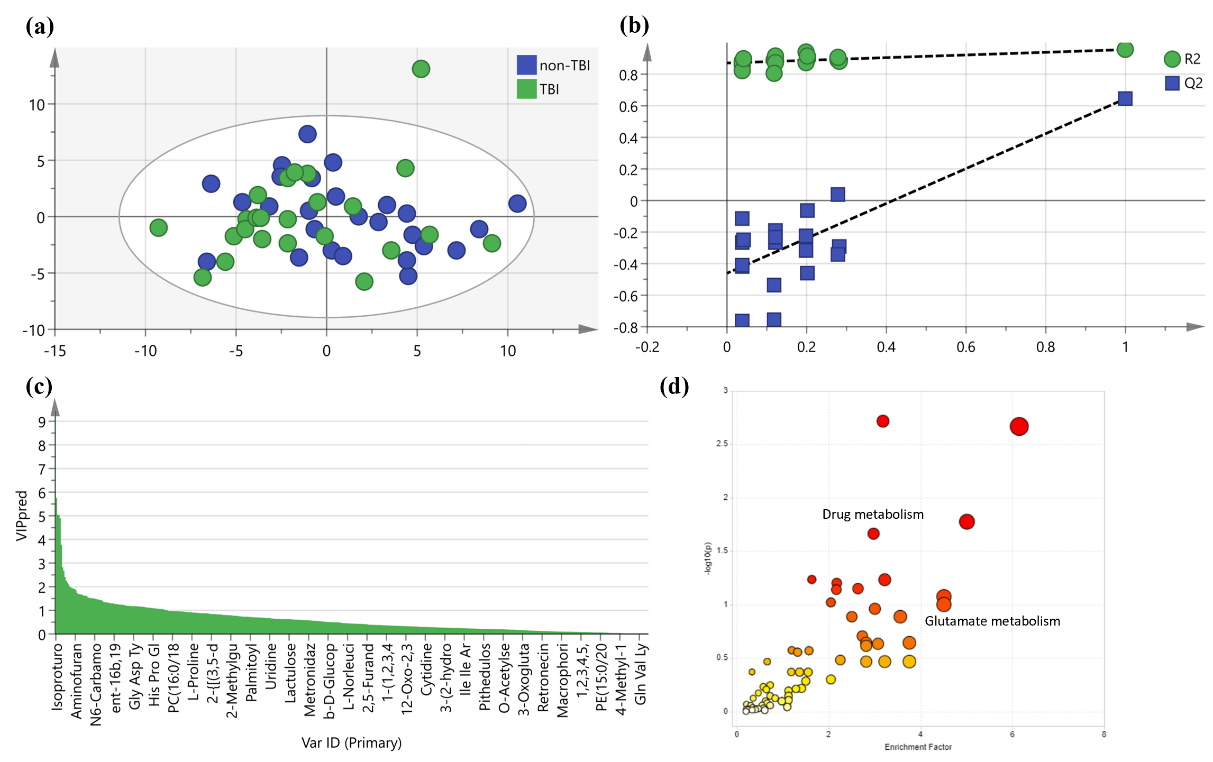


**Supporting Figure 2. Supplementary analysis of CSF metabolomic of TBI-induced DoC and non-TBI-induced DoC.** (a) Score plot of unsupervised PCA overview of CSF metabolic profiling of DoC patients with different etiologies; (b) Permutation testing of OPLS-DA model and the distributions of the R2/Q2-values; (c) VIP values of CSF metabolites; (d) Biological pathway of differential metabolites.


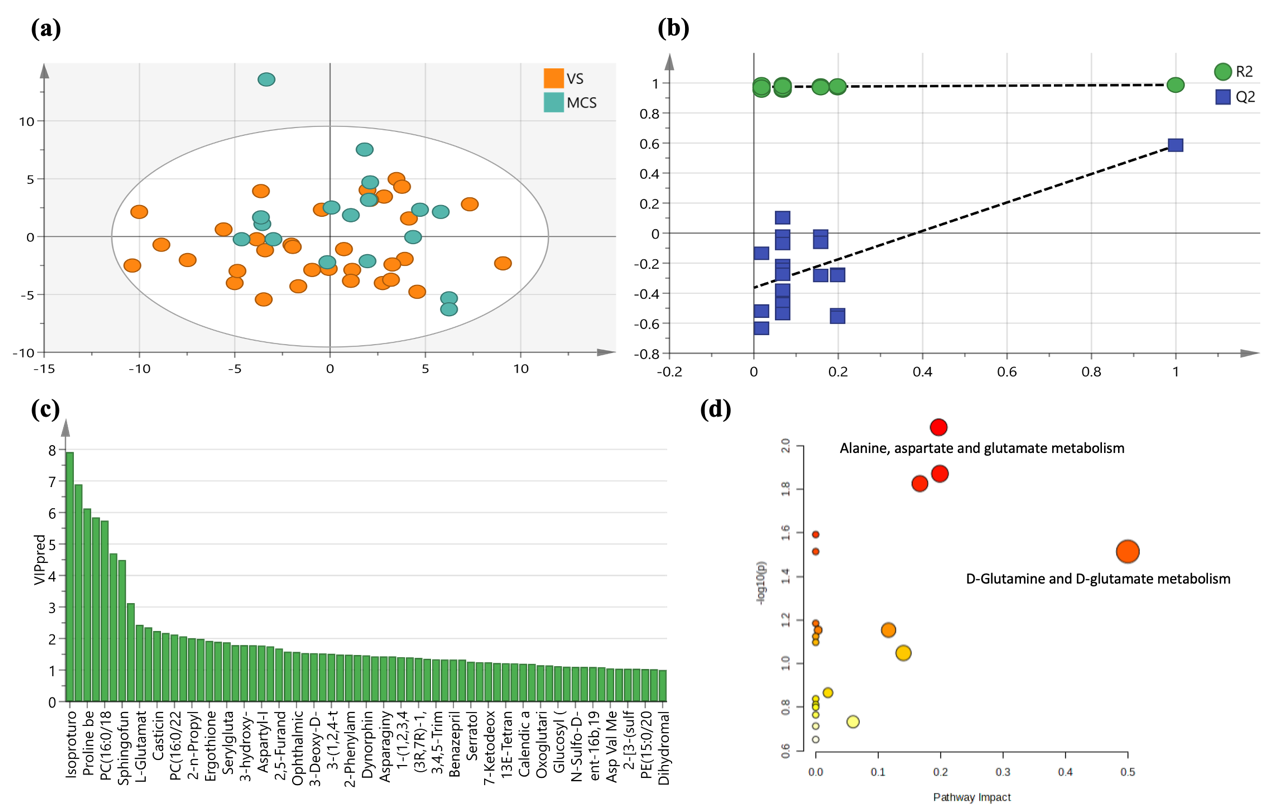


**Supporting Figure 3. Supplementary analysis of CSF metabolomic of DoC patients with different diagnosis.** (a) Score plot of unsupervised PCA overview of CSF metabolic profiling of patients with different CRS-R scores; (b) Permutation testing of OPLS-DA model and the distributions of the R2/Q2-values; (c) VIP values of CSF metabolites; (d) Biological pathway of differential metabolites.


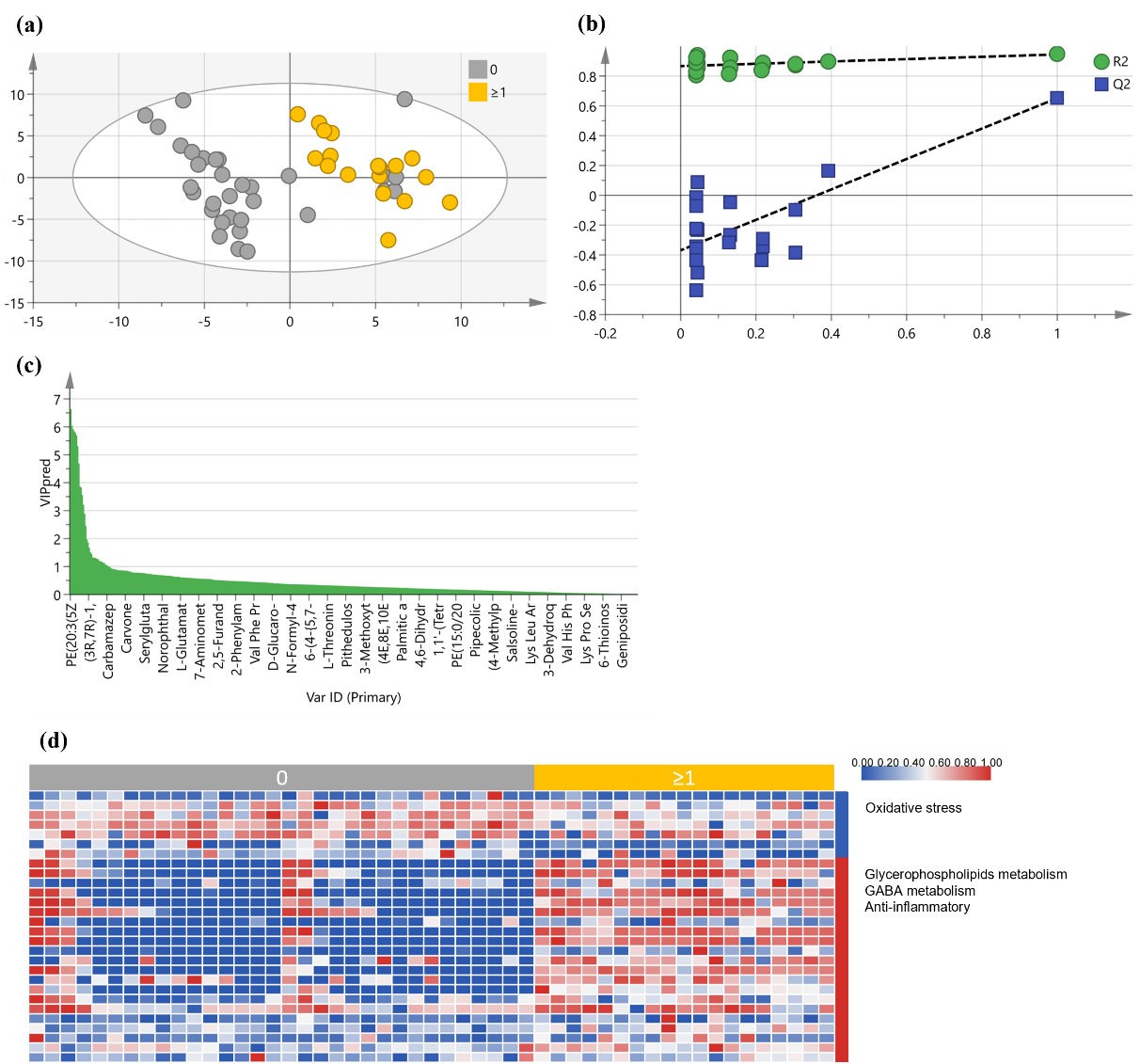


**Supporting Figure 4. Supplementary analysis of CSF metabolomic of DoC patients with different prognoses.** (a) Score plot of unsupervised PCA overview of CSF metabolic profiling of patients with different prognoses; (b) Permutation testing of OPLS-DA model and the distributions of the R2/Q2-values; (c) VIP values of CSF metabolites; (d) Heatmap of differential metabolites in the two groups and the differential metabolic pathways listed on the right side (blue indicate relative lower and red indicate relative higher in the group with better prognosis).
